# Unidirectional Photoreceptor-to-Müller Glia Coupling and Unique K^+^ Channel Expression in Caiman Retina

**DOI:** 10.1371/journal.pone.0097155

**Published:** 2014-05-15

**Authors:** Astrid Zayas-Santiago, Silke Agte, Yomarie Rivera, Jan Benedikt, Elke Ulbricht, Anett Karl, José Dávila, Alexey Savvinov, Yuriy Kucheryavykh, Mikhail Inyushin, Luis A. Cubano, Thomas Pannicke, Rüdiger W. Veh, Mike Francke, Alexei Verkhratsky, Misty J. Eaton, Andreas Reichenbach, Serguei N. Skatchkov

**Affiliations:** 1 Departments of Pathology, Biochemistry and Physiology, Universidad Central Del Caribe, Bayamón, Puerto Rico, United States of America; 2 Paul Flechsig Institute of Brain Research, Faculty of Medicine, University of Leipzig, Leipzig, Germany; 3 Division of Soft Matter Physics, Department of Physics, University of Leipzig, Leipzig, Germany; 4 Department of Physical Sciences, Universidad de Puerto Rico, Recinto de Río Piedras, Río Piedras, Puerto Rico, United States of America; 5 Department of Physiology, Development and Neuroscience, University of Cambridge, Cambridge, United Kingdom; 6 Charité, Berlin, Germany; 7 Translational Centre for Regenerative Medicine (TRM) University of Leipzig, Leipzig, Germany; 8 Faculty of Life Sciences, University of Manchester, Manchester, United Kingdom; Institut de la Vision, France

## Abstract

**Background:**

Müller cells, the principal glial cells of the vertebrate retina, are fundamental for the maintenance and function of neuronal cells. In most vertebrates, including humans, Müller cells abundantly express Kir4.1 inwardly rectifying potassium channels responsible for hyperpolarized membrane potential and for various vital functions such as potassium buffering and glutamate clearance; inter-species differences in Kir4.1 expression were, however, observed. Localization and function of potassium channels in Müller cells from the retina of crocodiles remain, hitherto, unknown.

**Methods:**

We studied retinae of the Spectacled caiman (*Caiman crocodilus fuscus*), endowed with both diurnal and nocturnal vision, by (i) immunohistochemistry, (ii) whole-cell voltage-clamp, and (iii) fluorescent dye tracing to investigate K^+^ channel distribution and glia-to-neuron communications.

**Results:**

Immunohistochemistry revealed that caiman Müller cells, similarly to other vertebrates, express vimentin, GFAP, S100β, and glutamine synthetase. In contrast, Kir4.1 channel protein was not found in Müller cells but was localized in photoreceptor cells. Instead, 2P-domain TASK-1 channels were expressed in Müller cells. Electrophysiological properties of enzymatically dissociated Müller cells without photoreceptors and isolated Müller cells with adhering photoreceptors were significantly different. This suggests ion coupling between Müller cells and photoreceptors in the caiman retina. Sulforhodamine-B injected into cones permeated to adhering Müller cells thus revealing a uni-directional dye coupling.

**Conclusion:**

Our data indicate that caiman Müller glial cells are unique among vertebrates studied so far by predominantly expressing TASK-1 rather than Kir4.1 K^+^ channels and by bi-directional ion and uni-directional dye coupling to photoreceptor cells. This coupling may play an important role in specific glia-neuron signaling pathways and in a new type of K^+^ buffering.

## Introduction

Müller glial cells [1] serve numerous fundamental functions in the retina of vertebrates; many of these functions depend on potassium channels, responsible for a high potassium conductance of the cell membrane [2], [3], [4]. Although the electrophysiological membrane properties, as well as the main functions, of Müller cells are similar among the vertebrates, distinct inter-specific differences have been observed even between closely related mammals such as monkeys and humans [5]. To further investigate Müller cells functional diversity, possibly reflecting adaptations to specific retinal circuits, it is desirable to study Müller cells from different groups of vertebrates. A wide variety of mammalian Müller cells have been investigated (e.g., [6]); as well as fishes (elasmobranchs and teleosts: [7], [8], [9] and amphibians (salamanders and anurans: [9], [10], [11], [12]. In reptilians, however, only Müller cells from the diurnal water turtle, *Pseudemys scripta elegans*, were characterized (e.g., [13], [14], [15], [16]). Here we report a study of Müller cells from retinae of caiman (*Caiman crocodilus fuscus*), which has perfect night vision as well as vision in the bright daylight, with a large scale of adaptation to different light intensities. This ability is reflected by several morphological and functional idiosyncrasies in the caiman vision system [17]. Incidentally, crocodiles are closer related to birds (in which Müller cells were never studied electrophysiologically) than to the turtles (e.g., [18], and references therein) which makes the caiman an even more interesting subject of examination.

Radially oriented Müller cells span the whole thickness of the retina and conduct light to photoreceptors [19]. These cells contact all neuronal elements located within the retina. Spatial buffering of extracellular K^+^ ions represents another most fundamental and extensively studied function of the Müller cell. In dark adapted retina, cells face large K^+^ gradients, with K^+^ concentrations ranging between 6–8 mM at the photoreceptor layer (i.e., at the distal part of Müller cell) and 2–3 mM at the vitreal surface where (i) Müller cell endfeet abut the vitreous body and (ii) complex ionic changes occur during light stimulation [20], [21], [22], [23]. Specific spatial distribution of K^+^ channels [24] allow Müller cells to redistribute K^+^ ions from sites of high extracellular concentration to ‘buffering reservoirs’ such as the vitreous fluid or the intraretinal blood vessels, and thus prevent elevations of extracellular K^+^ that may cause over-excitation of neurons with subsequent loss of information processing.

In the Müller cells and astrocytes of humans and of most animals studied, inwardly rectifying K^+^ (Kir) channels, specifically Kir4.1 (Kcnj10), play a key role for glia-neuron interactions (for recent reviews, see [3], [25], [26], [27]), being fundamental for example for glutamate clearance [28], [29]. Genetic variations of Kir4.1 channels in humans and animals underlie severe disorders in the brain and in the retina, such as epilepsy, disruption of electroretinogram, glaucoma, stroke, ataxia, hypokalemia, hypomagnesemia, and metabolic alkalosis [27], [30], [31], [32], [33]. In addition, recently identified Kir4.1 mutations were found to result in autoimmune inhibition, contributing to pathogenesis of multiple sclerosis [34], hearing loss [35], autism [36] and seizures [30], [33]. The mutated Kir4.1 protein is not inserted in the membrane or the channels are blocked, as revealed by the absence of representative potassium currents [37]. A loss of Kir4.1 channels function was also found in the retina and in the brain in diabetes [38], transient ischemia [39], and in trauma [40]; with deficit in Kir4.1 mediated permeability being linked to failures in neuronal function and neuronal cell death [3].

In Müller glia, Kir4.1 channels operate in concert with other types of K^+^ channels, which include (i) ATP-sensitive potassium channels (K(ATP)) assembled from Kir6.1 and SUR1 subunits [41], [42], [43], [44], [45], (ii) Kir2.1 inwardly rectifying channels [3], [24] and (iii) two pore domain (2P) potassium channels of the TASK family [43], [46], [47]. Among these channels the Kir2.x family displays strong inward rectification, and thus cannot mediate the K^+^ outward currents essential for spatial buffering [24]. The ATP-sensitive Kir6.x family, on the other hand, requires two preconditions to function [48], [49]. First, a functional K(ATP) channel can only be assembled from two structurally diverse proteins: the Kir6.x (Kir6.1 or Kir6.2) subunits and a sulfonylurea receptor (SUR) of the ATP-binding cassette superfamily (SUR1, SUR2A or SUR2B) [50], [51], [52]. Second, Kir6.1/SUR1 channels are inactivated at physiological ATP concentrations [45]; rather, a depletion of ATP is necessary to open these channels [53], [54], unless phospholipids such as PIP3, PIP2, PIP and PI partially remove the ATP block [54], [55]. In contrast, Kir4.1 channels are functional under normal conditions, and ATP is even necessary for their opening [56]. Finally, the TASK (KCNK family) channels contribute only minutely to the whole-cell currents recorded from frog and mammalian Müller cells [3], [47]. Thus, it is difficult to contemplate how these K^+^ channels may maintain the functions of Müller cells in the absence of Kir4.1 channels.

Surprisingly in the present study we found that Kir4.1 channels are absent in caiman Müller glial cells, which, however, express TASK channels. Furthermore, we observed a unique communication between photoreceptors and attached Müller cells, with uni-directional permeation of the fluorescent dye, sulforhodamine, from cones to Müller glial cells. We hypothesize that in the caiman retina, spatial K^+^ buffering may involve both Müller cells and photoreceptors via trans-cellular K^+^ fluxes. Preliminary results were reported in abstract form [57], [58].

## Materials and Methods

### Animals

Experiments were carried out with IACUC approval and in accordance with the ARVO Statement for the Use of Animals in Ophthalmic and Vision Research and according to institutional animal care and use guidelines. Adequate measures were taken to minimize pain or discomfort to experimental animals. Müller cells were isolated from retinae of adult caiman (*Caiman crocodilus fuscus*) as previously described [12], [45], [47].

### Immunohistochemistry

Retinal sections from 8 caimans were used for the immunohistochemical studies. Eyes were perfusion-fixed *in situ*. Caimans (with a length of 50 cm to 120 cm on average) were immobilized on ice before being anesthetized. Animals were subsequently i.p. anesthetized with tiletamine HCl/Zolazepam HCl, 5 mg/kg before intraventricular perfusion of fixatives. The thorax was opened and a catheter was placed in the left ventricular chamber of the heart for vascular perfusion. Perfusion for immunohistochemistry contained 4% paraformaldehyde (as the only fixative) in phosphate buffered solution (PBS): NaCl 136.9 mM, KCl 2.7 mM, Na_2_HPO_4_ 10.1 mM, KH_2_PO_4_ 1.8 mM with pH 7.4 for 30 minutes. The right atrium was cut to allow outflow of perfusate. After perfusion, eyes were enucleated and pieces (0.5 x 0.5 mm) of the retina were cut and rinsed in fresh PBS.

For agar-embedding we used the isolated retinal pieces embedded in 3% agarose (w/v) in PBS as previously described [39], [47] and agar cubes with tissue inside were cut by 80 µm sections. For cryo-embedding, after fixative perfusion the tissues were cryoprotected by immersion in 0.15 M sucrose in 0.1 M phosphate buffer, pH 7.4 (for 24 hrs), 0.5 M sucrose (for 24 hrs) and 0.8 M sucrose (for 48 hrs) and subsequently frozen at −60°C in liquid pentane and then stored in a −80°C freezer until next use. Cryostat sections of 25 µm cut using a vibratome (Leica VT1000S, Leica, Germany) were used.

For immunofluorescence analysis, retinal sections were incubated in 5% normal serum with 0.3% Triton X-100 and 1% dimethyl sulfoxide (DMSO) in PBS for 1 hr and subsequently overnight at 4°C with primary antibodies. After the washing of retinal slices from primary antibody in 1% bovine serum albumin in saline, the secondary antibodies were applied for 4 hrs at room temperature. Texas Red-conjugated donkey anti-goat IgG (1∶200), carbocyanin Cy5-coupled donkey anti-rabbit IgG (1∶200), Cy3-coupled goat anti-rabbit IgG (1∶200), Cy5-coupled goat anti-mouse IgG (1∶200), Cy2-coupled donkey anti-rabbit IgG (1∶200), and Cy2-coupled goat anti-rabbit IgG (1∶500; Dianova, Hamburg, Germany) were used. Controls were obtained by omitting the primary antibody. The images were obtained using confocal scanning microscopes (LSM 510 META, Zeiss, Oberkochen, Germany and Olympus Fluoview FV1200, Olympus, Japan).

We used the following primary antibodies: (i) for glial markers: mouse anti-S100β (1∶1000; Sigma-Aldrich, S2532), mouse anti-glial fibrillary acidic protein (GFAP; 1∶300; Sigma-Aldrich, G6171), mouse anti-glutamine synthetase (GS; 1∶500; Millipore, MAB302), mouse anti-vimentin (1∶300; PROGEN Biotechnik GmbH, Heidelberg, Germany, VIM 3B4), (ii) for channel proteins: rabbit anti-TASK-1 (1∶200; Santa Cruz Biotechnology, SC-28635), rabbit anti TREK-2 (1∶500; Alomone), rabbit anti TASK-3 (1∶300; Alomone), mouse anti-Kir4.1 (1∶300; Sigma-Aldrich, WH0003766M1), rabbit anti-Kir4.1 (1∶200; Alomone, APC-035), rabbit anti-Kir6.1 (1∶200; Alomone, APC-105), rabbit anti-Kir6.1, -Kir6.2, and -SUR1 (1∶200; 1∶150; 1∶200 respectively; R.W. Veh, Charité, Berlin), rabbit anti-aquaporin-4 (AQP4; 1∶200; Sigma-Aldrich, A5971), rabbit anti-aquaporin-4 (AQP4; 1∶200; Santa Cruz Biotechnology, SC-20812) and (iii) rabbit anti-G_αt1_ (G_α_ rod transducin; 1∶200; Santa Cruz Biotechnology, SC-389) for rod photoreceptor labeling.

For the detection of the gap junction protein connexin 43 (Cx43) we used rabbit anti-Cx43 (1∶100; Sigma-Aldrich, C6219), whereas for labelling glutamine synthetase mouse anti-glutamine synthetase (1∶1000; Millipore, MAB302) followed by the secondary antibodies Cy3-conjugated donkey anti-rabbit IgG (1∶200) and Cy5-conjugated donkey anti-mouse IgG (1∶200). To visualize the cone photoreceptors, i.e., their segments and their synaptic pedicles, we used the biotin-conjugated lectin peanut agglutinin (1∶200; PNA, Sigma-Aldrich, Germany) which specifically binds to cone photoreceptor cells [59]. During the incubation with the secondary antibodies, streptavidin labelled with Cy2 (1∶200; Sigma-Aldrich) was applied to bind to PNA. The cell nuclei were stained with Hoechst 33258.

In summary, the above described retinal markers were visualized using either the DAB procedure with nickel contrast (Kir4.1, Kir6.1, Kir6.2, SUR1, TASK-1, TASK-3, TREK-2) or a fluorescence method for laser scanning confocal microscopy (Kir4.1, GFAP, Vimentin, S100β, GS, AQP4, DAPI, Hoechst, PNA, G_αt1_ and Cx43). The sections were processed for immunohistochemical detection as previously described [44], [46], [47] or for Kir4.1, Kir6.1 and Kir6.2 channels [41], [45], [46], [60], [61].

### Electrophysiology in retinal wholemounts

Caimans were immobilized on ice and dark adapted for 1 hr before being anesthetized as described above. Anesthetized caimans were sacrificed by decapitation followed by removal of eyes. Retinae isolated from eyes were treated with a mix of collagenase/dispase (2 mg/mL) and DNase-1 (0.1 mg/mL) in PBS (pH 7.4) for 30 min at room temperature. After treatment, retinae were washed in PBS and placed in a recording chamber for electrophysiological recording of the vitreal endfeet of Müller cells. The extracellular solution (ECS) to perfuse retinal tissue contained (in mM): NaCl 110, CaCl_2_ 2, MgCl_2_ 1, NaH_2_PO_4_ 1.25, NaHCO_3_ 25, D-glucose 25, KCl varied from 2.5 to 10 mM (substituted by NaCl to adjust osmolarity); pH 7.4, after aeration by carbogen (95% O_2_+5% CO_2_). Electrodes were filled with intracellular solution (ICS) containing (in mM): K-gluconate 130, Na-gluconate 10, NaCl 4, HEPES 10, MgATP 4, phosphocreatine 4, NaGTP 0.3, pH adjusted to 7.2 with KOH/HCl. Spermine (0.25 mM; Sigma-Aldrich) was added to ICS in accordance with the finding that free spermine levels in Müller cells are in the submillimolar concentration range [62]. After filling with ICS, the micropipette resistance was ∼8 MΩ. Voltage and current clamp recordings in whole-cell patch-clamp mode were performed using a MultiClamp 700A patch-clamp amplifier with a DigiData 1322A interface (Molecular Devices, Inc., Sunnyvale, CA, USA). The cells were kept at holding potential equal to equilibrium resting potential (to keep membrane current at zero level) and the cells were stimulated by a step to −150 mV (for 120 ms) with following rising voltage ramp to +150 mV during 80 ms and then a step back to resting voltage. The pClamp 10 software package (Molecular Devices, Inc., CA) was used for data acquisition and analysis.

Note: retinal tissue stored for longer than 1 hr was not used. To study the coupling between Müller cells in retinal wholemounts, the fluorescent dye Lucifer Yellow (LY) 2 mM (Sigma-Aldrich) was added to the ICS as in [63].

### Electrophysiology in isolated cells

Retinae were cut into pieces (0.5 x 0.5 mm) and rinsed in fresh Ca^2+^-Mg^2+^-free PBS solution (osmolarity was adjusted to 309 mOsm which corresponds to the osmolarity measured for vitreal liquid sampled from caiman eyes), then were transferred into Ca^2+^-Mg^2+^-free PBS containing papain (24 unit/ml) for 30 min, at 37°C. After washing and trituration in PBS and then in Dulbecco's Modified Eagle medium (DMEM), isolated cells were briefly rinsed in DMEM containing 0.001 mg/ml DNase-1 (Sigma-Aldrich, D-4263, from bovine pancreas). The cells were then washed in DNase-free DMEM medium, stored for 10 min on ice for sedimentation; then the supernatant was exchanged for fresh DMEM and the cells stored on ice. This procedure yielded Müller cells with characteristic morphology and preserved fine processes. We investigated two types of isolated cells: single Müller cells (SM) and Müller cells with attached photoreceptors (SMP). Cells were placed in the recording chamber, allowed to settle on the bottom of the chamber mounted on the stage of an inverted microscope (Nikon DIAPHOT 300, Nikon, Japan) with attached three-axis water hydraulic fine micromanipulator (MHW-3, Narishige, Japan) or an upright microscope (BX51WI, Olympus, Japan) with piezoelectric micromanipulators (MX7500 with MC- 1000 drive, Siskiyou, Inc., Grants Pass, OR) used for positioning micropipettes during voltage-clamp and current-clamp recordings. The electrophysiological recordings were performed at room temperature. Electrodes for whole cell recording were pulled in four steps (using Sutter P-97 puller, USA) from hard glass (GC-150-10 glass tubing, Clark Electromedical Instruments, England). Electrodes were filled with intracellular solution (ICS) containing (in mM): KCl 130, MgCl_2_ 1, CaCl_2_ 1, EGTA 10, HEPES 10, Na_2_ATP 3, spermine HCl 0.25, pH adjusted to 7.2 with KOH/HCl. They had resistances of 4-6 MOhm; after cell penetration, the access resistance was 10–15 MOhm, compensated by at least 75%. The extracellular solution (ECS) contained (in mM): NaCl 145, CaCl_2_ 2.5, MgCl_2_ 2, and HEPES 10; KCl varied from 2.5 to 10 mM (substituted by NaCl to adjust osmolarity). For recording from single isolated cells we used an Axopatch-200B amplifier with a CV-203BU headstage and a MultiClamp 700A amplifier with CV-7A headstage. DigiData 1200A and DigiData 1322A interfaces were used respectively for data acquisition and analysis (Axon Instruments, Molecular Devices, USA). High frequencies >1 kHz were cut off and signals were digitized at 5 kHz. The pCLAMP-9 (Axon Instrument, USA) and pCLAMP-10 (Molecular Devices, USA) software packages were used for data acquisition and analysis.

To isolate conductances mediated by different potassium channels we used established pharmacological procedures: channels of TASK family were blocked by bupivacaine 0.1–1 mM [47], [64], whereas Kir channels were blocked by 0.1–0.3 mM of barium [12], [65], [66]. ATP (3 mM) was used in the ICS to inhibit Kir 6.1/SUR1 (K(ATP)) channels [45]. Spermine (250 µM) was added to the ICS to block the outward component of Kir channels [67], [68]. Addition of spermine in the ICS also helped to separate TASK-mediated currents from Kir currents [47]. At the concentrations used, barium has little effect on TASK channel currents [69], [70], [71]. The patched cells were kept at holding potential equal to equilibrium resting potential and then a step to −150 mV (for 10 ms) with following rising voltage ramp to +150 mV (for 16 ms) and back to resting voltage. To ensure the ionic nature of whole cell currents, we performed standard control experiments where KCl was substituted with CsCl in the ICS and in ECS; under these conditions, no currents were recorded (not shown). Inhibitors of Kir and 2P-domain channels, barium chloride and bupivacaine were purchased from Sigma-Aldrich Chemical Co. (St. Louis, MO, USA and Taufkirchen, Germany).

### Fluorescent tracer diffusion studies

We analyzed the diffusion of the gap-junction channel-permeable dyes LY (see above) and sulforhodamine-B, dialyzed in whole-cell configuration in current clamp (zero current) mode. The patch pipettes were filled with ICS described above and containing 2 mM of LY or sulforhodamine-B. LY (Sigma-Aldrich, MW 457.25), is a green- to yellow-fluorescent negatively charged (-2 charges) dye, while sulforhodamine-B (Molecular Probes, MW 558.66) is an orange- to red-fluorescent non-charged water-soluble (polar) sulfonic acid tracer with strong absorption and good photostability [63], [72], [73], [74], [75].

### Data Analysis

Data were analyzed using pCLAMP-10 (Molecular Devices, USA), Origin 8 software (OriginLab, Northampton, MA, USA) and are reported as mean ± standard error of the mean. Significant differences between groups of data were evaluated using Student's t-test.

## Results

### Glial markers in caiman Müller cells

**Figure 1:**
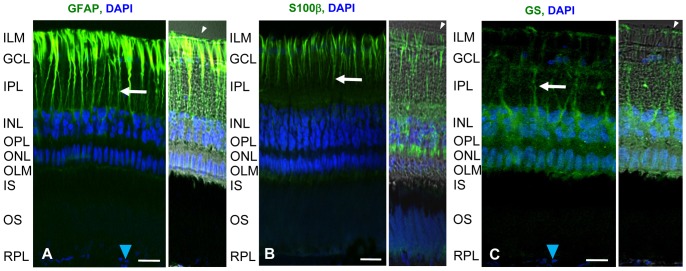
Expression of retinal glial and photoreceptor markers in the caiman retina. Immunostaining for glial fibrillary acidic protein (GFAP), glial specific calcium binding protein (S100β), glutamate-to-glutamine converting enzyme glutamine synthetase (GS), and DAPI (blue) which shows nuclei of retinal cells. (**A**) Confocal (left) and Nomarski (right) images showing the expression of GFAP, (**B**) S100β, and (**C**) glutamine synthetase. The markers delineated the overall structure of Müller cells: white arrowheads point to the inner limiting membranes (ILM) in the vitreal endfeet areas. White arrows point to stalks of Müller cells. Blue arrowheads show nuclei of pigment epithelium cells. Scale bar  = 20 µm. ILM-inner limiting membrane, GCL-ganglion cell layer, IPL-inner plexiform layer, INL-inner nuclear layer, ONL-outer nuclear layer, OPL-outer plexiform layer, IS-inner segments of photoreceptors, OS-outer segments of photoreceptors, RPL-retinal pigment epithelium layer.

**Figure 2:**
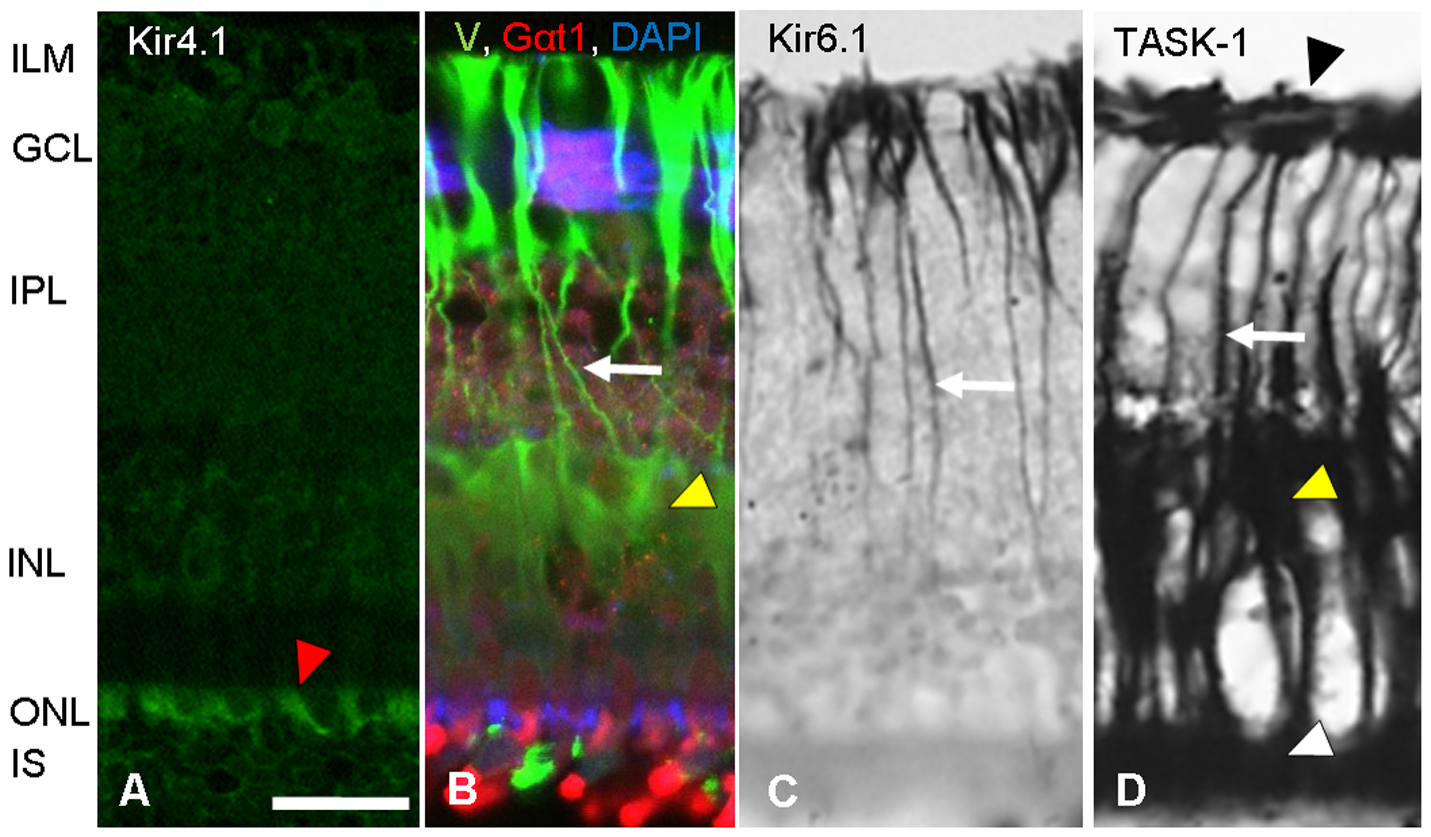
Differential localization of potassium channels and glial markers in the caiman retina. (**A**) Inwardly rectifying potassium channels Kir4.1 (green) are localized in the area of the photoreceptors and in outer nuclear layer (red arrowhead), but not in Müller cell processes such as stalks or endfeet. This is different from most of vertebrates where Kir4.1 channels were found in Müller cells [3]. (**B**) Glial Müller cell specific marker vimentin, V (green); photoreceptor specific alpha-1 subunit of transducin, Gαt1 (red); nucleus-specific marker, DAPI (blue). Vimentin staining is observed in endfeet, somata, stalks and in distal processes (yellow arrowhead points somata, white arrow shows stalk). DAPI staining (blue) shows nuclei of retinal cells. Alpha-1 subunit of transducin (Gαt1, red) is a marker of the second messenger G-protein cascade and it is found mostly in photoreceptors. (**C**) ATP-dependent K^+^ channel, Kir6.1 (black), is localized in stalks (white arrow) and in endfeet, while (**D**) two pore domain acid sensitive K^+^ channels, TASK-1 (black) are found in whole Müller cell compartments: in endfeet (black arrowhead), stalks (white arrow), somata (yellow arrowhead) and in distal processes (white arrowhead). Scale bar  = 20 µm. ILM-inner limiting membrane, GCL-ganglion cell layer, IPL-inner plexiform layer, INL-inner nuclear layer, ONL-outer nuclear layer, IS- inner segments of photoreceptors.

First, we analyzed the presence of the established glial marker proteins, S100β (glial specific calcium binding protein), GS (glutamine synthetase), vimentin, and GFAP (glial fibrillary acidic protein) in caiman retinal Müller cells. In addition, we examined the localization of the alpha subunit of G-protein transducin (Gαt1), a G-protein involved in phototransduction, in caiman photoreceptor cells. Immunoreactivity for all four glial proteins was identified in caiman Müller cells, and Gαt1 was detected in caiman photoreceptor cells (Figs. 1, 2).

We found a robust expression of GFAP in all Müller cells, in somata, in processes and even in very fine distal branches (Fig. 1A). S100β (Fig. 1B) and vimentin (Fig. 2) were expressed throughout the length of all Müller cells, in all retinal areas. Prominent S100β and vimentin staining was observed in somata, distal processes and endfeet. Similarly, GS immunoreactivity was observed in all Müller cell compartments including somata and distal processes (Fig. 1C). In many vertebrate species, strong GFAP expression is only observed in cases of reactive gliosis whereas normal Müller cells are devoid of GFAP immunolabeling (e.g., [3]). However, exceptions have been described; for instance, in the retina of goldfish [76] and horse [6], the Müller cells are GFAP immunopositive under normal conditions. Thus, the GFAP expression of caiman Müller cells is suggested here to represent a species-specific feature, rather than a pathological event.

It has been previously demonstrated that the water channel AQP4 is mainly localized in the endfeet of Müller cells within the rat retina [3]. Using different primary antibodies, AQP4 staining was found basically at the inner limiting membrane which abuts to the vitreal Müller cell endfeet (figure S1).

### Expression of Kir4.1, Kir6.1, Kir6.2 and SUR1

In all vertebrate species studied so far, Kir4.1 channels were found to be robustly expressed by Müller cell endfeet at the inner limiting membrane, and in ‘en-passant endfeet’ around blood vessels [3], [24], [44], [77], [78], [79], [80]. Most strikingly, Kir4.1 immunolabel was not located to caiman Müller cells (Fig. 2A). Rather, the channel protein was located to photoreceptor cells (Fig. 2A) similar as Gαt1 (Fig. 2B). Using different antibodies for Kir4.1 (and DAB versus fluorescent techniques) we found similar localization of Kir4.1 protein (DAB images not shown). Thus, immunoreactivity for Kir4.1 was co-localized with the photoreceptor marker, Gαt1 (Fig. 2B).

Immunoreactivity for Kir6.1 was found in caiman Müller cells (Fig. 2C) whereas Kir6.2 was located to neurons (not shown). This differential localization has also been observed in amphibians and in guinea pig [43], [44], [45]. However, it has been shown that Kir6.1 expression alone is not sufficient to mediate K^+^ currents; the sulfonylurea receptor subunit SUR1 is necessary as a partner of Kir6.1 to form functional K(ATP) channels in Müller glial cells [42], [45], in other glial cells [41] and in non-glial cells [48]. The SUR1 immunolabelling was not detected in caiman Müller cells (figure S2). Together, these data imply that, unlike in other vertebrate species studied so far, in caiman Müller cells the inwardly rectifying K^+^ channels, Kir6.1 and Kir4.1, are not available to mediate K^+^ currents, and to maintain the membrane potential of the cells, therefore, we studied other K^+^-channel localization.

### Localization of two pore domain K^+^ (2P) channels TASK-1, TASK-3, and TREK-2

To examine the putative presence of other potassium channels necessary to maintain the membrane potential of the cells [70] we used antibodies against three 2P-domain channels (TASK-1, TASK-3, TREK-2). Both TASK-1 and TASK-3 immunolabelling was detected in Müller cells, albeit with different intensity. Immunoreactivity for TASK-1 was strong; it was localized throughout the length of Müller cells, in endfeet, inner stem processes, somata, and distal processes (Fig. 2D); in contrast TASK-3 was rather weakly expressed only in distal processes of Müller cells (unpublished data). This pattern of TASK-1 expression throughout the Müller cells corresponds to what was reported for amphibian [46] and mammalian retinal Müller glia [47]. TREK-2 immunoreactivity was not detectable in caiman Müller cells (not shown). In summary, TASK-1 appears to be the dominant K^+^ channel type in caiman Müller cells. Further electrophysiological recordings were made to prove this assumption.

### Electrophysiological recordings from Müller cell endfeet in retinal wholemounts

**Figure 3:**
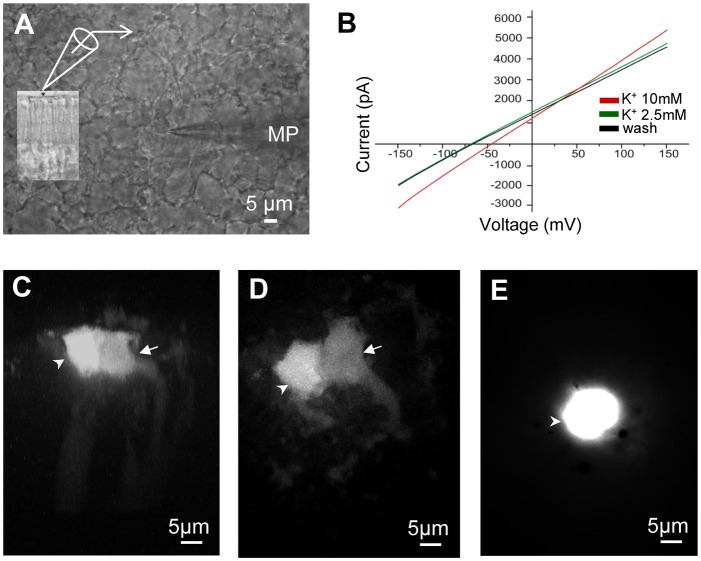
Müller cells in retinal wholemounts. (**A**) Recordings from vitreal endfeet of Müller cells. A patch pipette (MP) at the vitreal surface penetrating a single endfoot while many round- shaped endfeet with different diameters are visible. The patch clamp technique was used for recording the electrical currents and voltages as well as for fluorescent dye injection. The insert shows the differential interference contrast (DIC) image of a retinal axial section with a patch pipette positioned at the endfoot membrane. (**B**) Representative currents from caiman Müller cell endfeet in response to a voltage ramp of 80 ms duration from −150 mV to +150 mV (I/V-curves). The curves show the I/V relationship in different extracellular [K^+^], 2.5 mM and 10 mM, and the washout in 2.5 mM demonstrating classical glial sensitivity to potassium. (**C**) and (**D**) Two representative examples for Lucifer Yellow diffusion from injected endfoot (white arrowhead) to neighbor endfoot (white arrow) under control conditions. (**E**) Lucifer Yellow injection into a single endfoot in a retinal wholemount perfused with the gap junction blocker carbenoxolone (200 µM). The dye is not propagating to the neighboring cells.

In wholemount preparations of the caiman retina (Fig. 3A), Müller cells were patched at their endfeet at the top of the retina with photoreceptor side down. The average membrane potential (n  = 23 cells) was −69.8±1.3 mV. Membrane potential was highly sensitive to extracellular K^+^, with an average depolarization of 26.5±2.0 mV (n = 15) in response to [K^+^]_o_ changes from 2.5 mM to 10 mM, close to the Nernstian shift for K^+^ (∼29 mV) (Fig. 3B), whereas the current-voltage curves in 2.5 mM as well as 10 mM [K^+^]_o_ showed linear properties.

Neither barium (Ba^2+^), an efficient Kir channel blocker [12], [16], [65], [66], nor bupivacaine (a 2P-domain channel blocker, specific for TASK-1: [47], [64]) had a strong effect on glial membrane potential in the wholemount preparations; the depolarization caused by barium was 0.73±0.14 mV (n = 7) and the depolarization by bupivacaine was 0.56±0.32 mV (n = 7) (not shown).

Müller cells in the wholemount preparations can retain inter-cellular coupling thus affecting the space clamp [81]. To access the coupling in the wholemount preparation we injected the fluorescent dye, Lucifer Yellow, into the endfeet of the patch-clamped cells. After removing the pipette the dye was found also in adjacent Müller cells (Fig. 3C, D), indicating cell-cell coupling. This coupling was not extensive, as in most of the cases only one additional cell was labeled; a similar weak coupling has been described to occur between rabbit retinal Müller cells [82]. When the gap junction blocker carbenoxolone (200 µM) was applied, the dye remained in the injected cell (Fig. 3E) and no dye-coupling was observed. Together, these data show that whole-cell currents cannot be reliably recorded from the cells in the wholemount condition. To overcome this problem, we studied enzymatically dissociated single cells.

### Electrophysiological recordings from isolated caiman Müller cells

**Figure 4:**
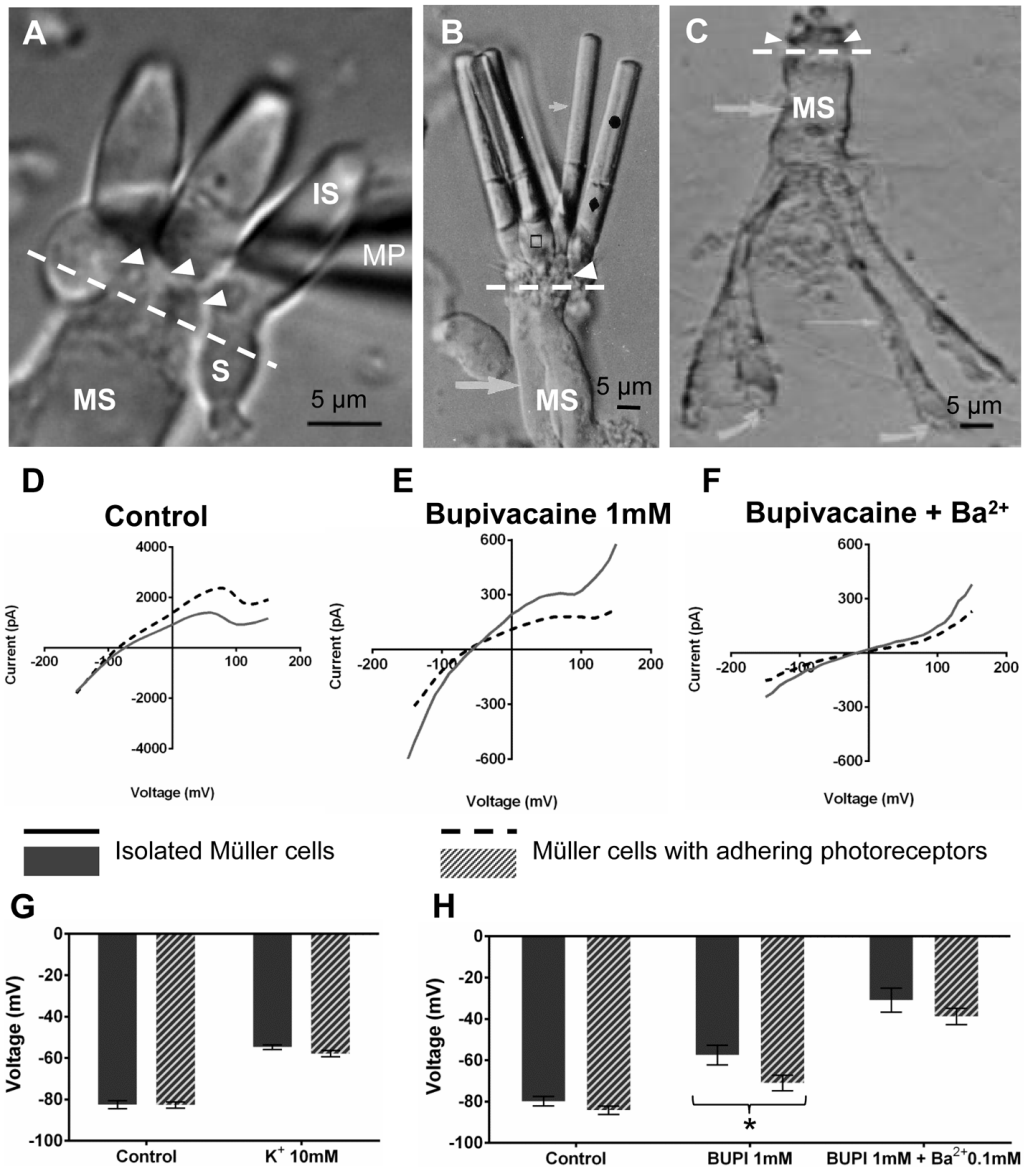
Cellular tandem between Müller cells and photoreceptors: involvement of 2P domain K^+^ channels. (**A**) Müller cell soma (MS) with attached cones approached by a patch pipette (MP). In **A**, **B**, and **C**, white arrowheads and white dotted lines show the area of contact between the inner segment (IS) of photoreceptors and MS. S points to the soma of cones. (**B**) Rods with outer segments (black circle), inner segments (black diamond) and somata (open black square) attached to MS. In **B** and **C**, thick white arrow shows MS. (**C**) Isolated Müller cell without photoreceptors. Curved arrows show endfeet. Thin white arrow shows stalk. (**D**) Average currents recorded from MS of isolated Müller cells (solid line, n = 5) and recorded from MS of isolated Müller cells with photoreceptors attached (dotted line, n = 5). The I/V-curves were obtained in response to a linear voltage ramp from −150 mV to +150 mV in control ECS K^+^ = 2.5 mM. Müller cells have linear outward currents near K^+^-equilibrium potential. (**E**) Effect of 2-P domain channel blocker bupivacaine (BUPI 1 mM) on isolated Müller cells with (dotted line) and without photoreceptors attached (solid line). After BUPI, residual currents are reduced ∼15 times. (**F**) Adding the Kir channel blocker barium (Ba^2+^, 100 µM) to BUPI, caused a near complete block of residual current (from −100 mV to +50 mV). (**G**) Membrane potentials of cells in 2.5 and 10 mM K^+^ ECS. [K^+^]_o_ = 10 mM induces depolarization. (**H**) Application of BUPI 1 mM and BUPI 1 mM with barium 100 µM were used to test for 2P-domain and Kir channels respectively. Single Müller cells (solid black column, n = 8) and cells with photoreceptors attached (grey column, n = 10) show different sensitivity to BUPI. Addition of Ba^2+^ further depressed membrane potentials in both cells, but with no significant difference. Error bars represent standard errors of the mean (SEM), where p<0.05 was considered significant (*).

Enzymatic dissociation yielded either completely or incompletely isolated (Fig. 4A, B, C) Müller cells. In the latter case, small numbers of cones (Fig. 4A) or rods (Fig. 4B) remained attached to a Müller cell. Whole-cell patch clamp recordings were made from completely isolated Müller cells (SM) as well as from Müller cells with adhering photoreceptor cells (SMP). The patch pipette was placed at the soma of the Müller cell if not stated otherwise.

Resting membrane potential in isolated Müller cells was more hyperpolarized than when recorded from endfeet in the retinal wholemounts. The average membrane potential recorded from SM in control ECS (K^+^ 2.5 mM) was −82.50±1.93 mV (n = 8) and SMP cells showed −84.20±2.03 mV (n = 10). Under control conditions membrane potentials were very similar between the SM and SMPs (Fig. 4). Thus, the measured resting membrane potentials were close to the ideal (Nernstian) K^+^ equilibrium potential, similar as reported previously from fish, amphibian, reptilian and mammalian Müller cells [3], [12], [16], [65], [66], [83], [84]. In addition, we tested cell responses to elevation of extracellular K^+^ from 2.5 mM to 10 mM; SM depolarized from −82.50±0.31 mV (control) to −54.75±1.11 mV (high K^+^; n = 8), (Fig. 4G). Similar depolarizations occurred in SMP, from −84.20±2.03 mV (control) to −57.90±1.46 mV (n = 10). If the Müller cells were patched at their endfeet, nearly identical values were measured.

Under bupivacaine, SM cells were depolarized from −79.86±2.27 mV to −57.50±4.72 mV (n = 8), whereas SMP cells were depolarized significantly less, from −84.20±2.03 mV to −71.00±3.76 mV (n = 10) (Fig. 4H). This difference in sensitivity to the TASK-1 blocker between SM and SMP suggests that the attached photoreceptors (Fig. 4A, B) may modify the Müller cell electrophysiology via cell-cell coupling. Bupivacaine (1 mM) reduced the membrane currents evoked by voltage ramps in voltage-clamp mode (Fig. 4D, E). I/V-curves after the bupivacaine block show a minor remnant of bupivacaine-insensitive Kir currents. The addition of Ba^2+^, a K^+^ channel blocker particularly effective for Kir channels, should thus reduce the difference in depolarization between SM and SMP cells. In fact, if Ba^2+^ (0.1 mM) was applied together with bupivacaine (1 mM) the cells were further depolarized (Fig. 4F, H); SM cells to −30.88±5.83 mV (n = 8) and SMP cells to −38.80±3.90 mV (n = 10).

Taken together, isolated Müller cells showed a nearly perfect K^+^-sensitivity and also a sensitivity to blockers, unlike cells patched in retinal wholemounts (cf. Fig. 3); this is ascribed to imperfect space clamp control in the wholemount situation. The electrophysiological data from isolated cells support the immunohistochemical data by revealing bupivacaine-sensitive currents that may be ascribed to functional TASK-1 channels. Noteworthy, however, in cells with adhering photoreceptors the depolarization induced by bupivacaine was smaller than in single Müller cells, suggesting that Kir channels contribute to the membrane potential (Fig. 4H). This could be explained if Müller cells and photoreceptor cells were electrically coupled. Thus, we tested the isolated cell groups (Fig. 4A, B) for a possible dye coupling.

### Coupling between cone and Müller cell

**Figure 5:**
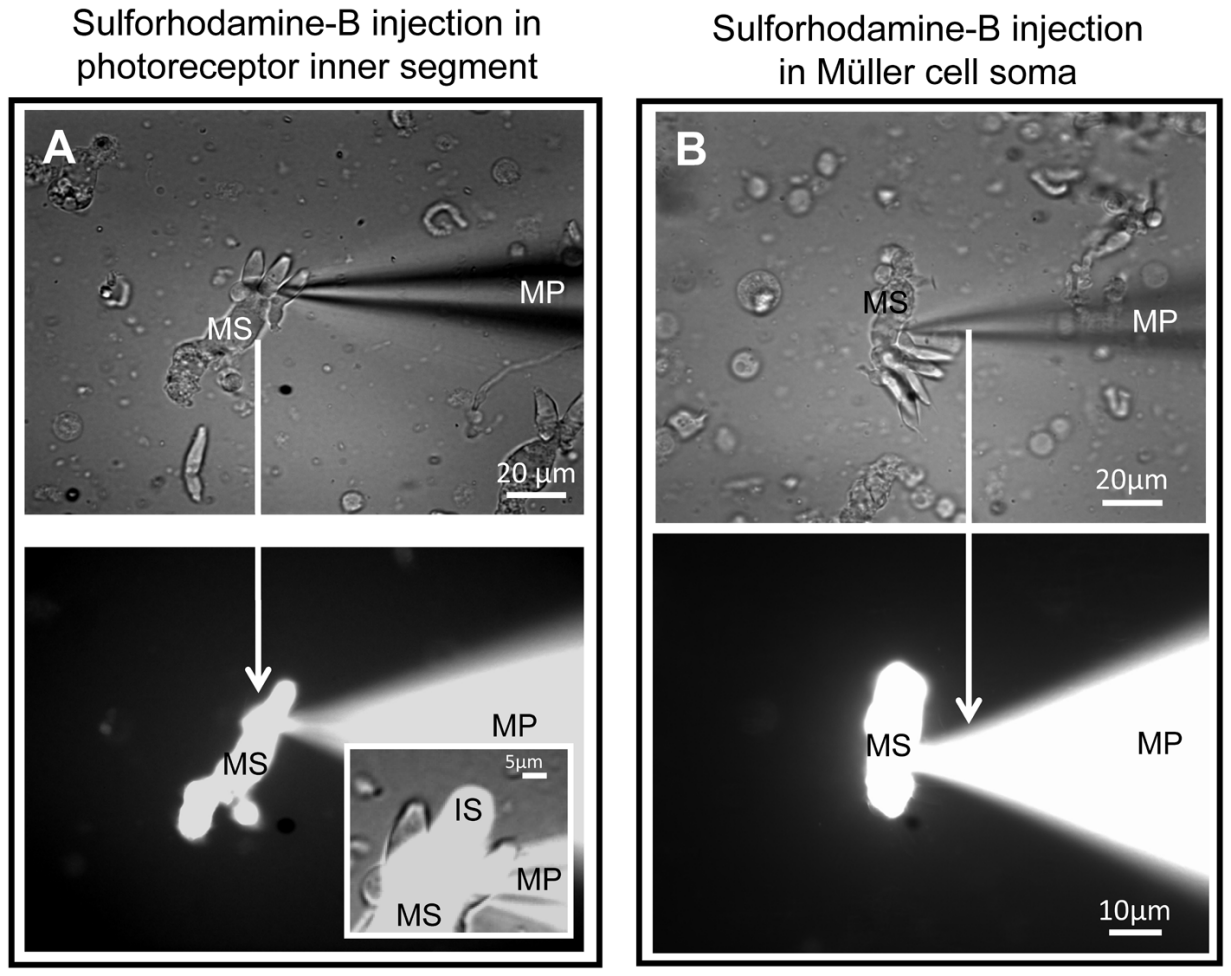
Unidirectional flow of sulforhodamine-B dye from cone to caiman Müller cell. (**A**) Upper panel: whole-cell patch clamp technique using micropipette (MP) filled with sulforhodamine-B penetrating the inner segment of a cone attached to a Müller cell. The dye filled the cone and the Müller cell: tracing of the photoreceptor cell body and inner segment (IS) and spreading throughout the whole Müller cell from the soma (MS) to the endfeet. Lower panel: combined (DIC and fluorescent) image showing that sulforhodamine-B is not permeable to neighboring photoreceptors, but filled only the cone which was patched. Insert shows an enlarged image of fine attachments of three cones to a single glial cell where two cones are not showing fluorescent dye. (**B**) Upper panel: whole-cell patch clamp of a Müller cell resulted in the dye-tracing of the cell body and endfeet, while no spreading of the dye occurred to the photoreceptors attached to this Müller cell. Lower panel: patch of the Müller cell soma only traced the Müller cell; the dye did not spread to the attached photoreceptors.

The electrophysiological and pharmacological data (Fig. 4D-H) suggest that Müller cells can transfer K^+^ from and to adhering photoreceptors, via a coupling between the Müller cell cytoplasm and that of the photoreceptor cells. We, therefore, analyzed the diffusion of the gap-junction channel-permeable dye sulforhodamine-B. The dye was applied intracellularly in whole-cell configuration when holding the cell in the current clamp mode. We detected the dye-coupling in SMPs; however, the spread of the tracer is unidirectional, occurring only in the direction from the cone to the Müller cell (Fig. 5A, B). When we patched the soma of a Müller cell the tracer easily filled the entire Müller cell body, but never spread to the photoreceptors (Fig. 5B). When patching the inner segment of one of the adhering cones, the tracer diffused throughout the photoreceptor and through the entire adjacent Müller cell (Fig. 5A). We also used several other dyes such as Lucifer yellow, Alexa Fluor-488, Alexa Fluor-568, but these compounds were not permeable from one cell to another in any direction (data not shown). This difference may be caused by the fact that Lucifer yellow and both Alexa dyes are negatively charged molecules whereas sulforhodamine-B is a neutral molecule. When a cone inner segment was patched, the tracer spread throughout the entire photoreceptor and the attached Müller cell (Fig. 5A), but not into neighboring photoreceptors (Fig. 5A, insert). Thus, the observed uni-directional tracer diffusion from a cone to a Müller cell but not to other adjacent cones, and not from the Müller cell to a cone represents a unique cell-to-cell communication for relatively large, uncharged molecules. By contrast, small cations such as K^+^ can probably permeate in both directions, as indicated by the electrophysiological data. We assumed that the movement of ions and sulforhodamine-B might be mediated by gap junctional coupling. To clarify this point, we performed an immunohistochemical staining for connexin 43 (Cx43).

### Cx43-immunohistochemistry

**Figure 6:**
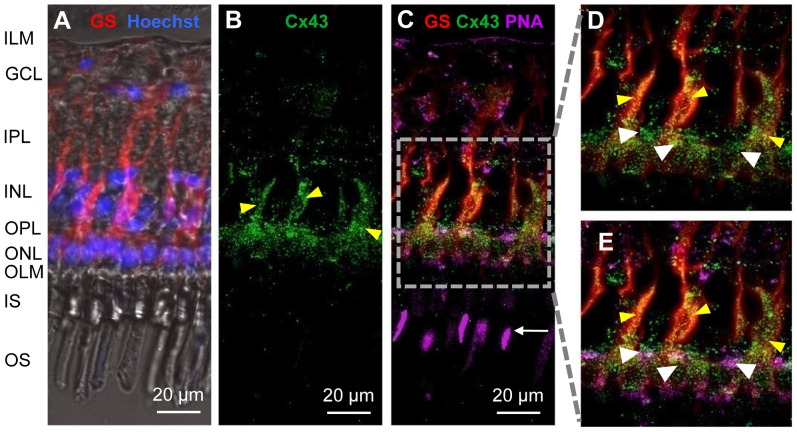
Localization of connexin 43 (Cx43) in the caiman retina. (**A**) Immunolocalization of the Müller cell-specific protein glutamine synthetase (GS, red) and staining of the nuclei by Hoechst 33258 (blue). The micrograph shows the overlay of the fluorescence image and the transmitted light with visible retinal layers. (**B**) Immunostaining of Cx43 (green), yellow arrowheads point to Müller cell-like structures. (**C**)-(**E**) Overlay of Cx43 and GS staining clearly demonstrates co-localization in Müller cell processes (yellow arrowheads). Moreover, cone outer segments and cone pedicles are stained by the lectin peanut agglutinin (PNA, purple). Localization of Cx43 in cone pedicles is demonstrated at higher magnification in (**E**) (white arrowheads). ILM-inner limiting membrane, GCL-ganglion cell layer, IPL-inner plexiform layer, INL-inner nuclear layer, OPL-outer plexiform layer, ONL-outer nuclear layer, OLM-outer limiting membrane, IS-inner segments of photoreceptors, OS-outer segments of photoreceptors.

In a study on rabbit retina, Müller cells were found to express Cx43 immunoreactivity [82]. Antibodies directed to Cx43 revealed robust, punctate labeling mainly in the outer caiman retina (Fig. 6B). Double-labeling of Müller cells by antibodies against glutamine synthetase (GS) and of cone photoreceptors by the lectin peanut agglutinin (PNA) showed that much of the Cx43 immunoreactivity can be ascribed to Müller cells and cone pedicles (Fig. 6).

## Discussion

### Unique distribution of glial K^+^ channels in caiman Müller cells

Here we provide the first morphological and functional characterization of Müller glial cells of the Spectacled caiman (*Caiman crocodilus fuscus*). Müller cells from crocodiles have been studied morphologically [85], [86], [87], [88], [89] but studies of the physiological characteristics of these cells were not yet performed. We show here that caiman Müller cells resemble Müller cells in some other vertebrate species by expressing classical glial markers, such as S100β, glutamine synthetase, GFAP (Fig. 1), and vimentin (Fig. 2B). Caiman Müller cells display a very negative membrane potential, close to the equilibrium potential for K^+^, similarly to that found in Müller cells of all other vertebrates studied so far (e.g, [3], [4]), and their membrane potential was sensitive to small changes of K^+^ concentration (Fig. 4G). However, we detected a striking peculiarity in the type(s) of K^+^ channels mediating this K^+^ conductance.

High K^+^ conductance of Müller cells of amphibian, reptilian and mammalian species has been well documented [3], [12], [16], [66], [90], [91], [92]. The expression of Kir4.1 (Kcnj10) in Müller cells seems to be much conserved among adult vertebrates [3]. There are two instances when Kir channels in Müller cells and other glia are not functional: (i) during very early development up to 10–15 postnatal days in Müller cells [91], [93], [94] and in astrocytes [95], [96], or (ii) in adulthood under pathological conditions when Kir4.1 may be mislocated and functionally inhibited [39], [60], [61], [84], [37], [97], [98]. In this study we used healthy adult animals.

We provide several lines of evidence suggesting that unlike in Müller cells of other healthy vertebrates, functional Kir-type K^+^ channels are not predominantly expressed in normal adult caiman Müller cells. First, Kir4.1 and Kir6.2 immunoreactivities were not detectable in caiman Müller cells. Second, the glial K(ATP) channel subunit, Kir6.1, was immunolabeled in the Müller cells but was not accompanied by its functionally essential SUR1 satellite subunit, [41], [44], [45]. Moreover, in the presence of ATP (in cytoplasm as well as in recording pipettes) the opening probability of Kir6.1 channels is extremely low [45], [54]. In addition, the I/V-curves recorded from enzymatically isolated single Müller cells (Fig. 4D, E) failed to display a Kir-like pattern (i.e., strong inward rectification near K^+^-equilibrium potential [67]). Therefore, Kir-type K^+^ channels known to occur in Müller cells (and other glial cells) of other vertebrates are not available in caiman Müller cells; this suggests that other types of K^+^ channels are responsible for maintaining the hyperpolarized membrane potential of these cells.

We found that TASK-1 2P channels were immunolocalized in Müller cells but not in other cells of the caiman retina (Fig. 2D). Membrane currents mediated by these channels are characterized by linear (or slightly rectified due to internal sodium block [99]) I/V-curves typical for TASK channels [64], [70], [99] that are expressed specifically in Müller glia of different animals [47]. This fits to our observation that the I/V curves in caiman Müller cells were linear near equilibrium potential (Fig. 4D). Furthermore, a TASK blocker bupivacaine [47], [64] inhibited outward currents almost completely (from 2.3 nA to ∼0.15 nA at +75 mV (Fig. 4D, E, dotted lines)). This indicates that TASK channels generate most of the K^+^ currents in caiman Müller cells. It cannot be excluded that there are other (hitherto unknown) K^+^ channels which are maintaining a (depolarized but substantial) membrane potential after Ba^2+^ and bupivacaine block (Fig. 4H) but certainly they cannot mediate large K^+^ currents (Fig. 4F). The location of aquaporin-4 (AQP4) in the caiman retina can be sufficient for water transport. AQP4 has been found in the endfeet processes at the (i) ILM and (ii) around blood vessels in the vascular retina [100]. However, the caiman has an avascular retina with no vessels in the retinal parenchyma, and as we demonstrated with two different antibodies, AQP4 is localized only in the endfeet area of the ILM, where TASK1 is located as well (Fig. S1). This opens the questions: Is an AQP4/TASK1 assembly functional for water transport as has been shown for AQP4/Kir4.1 [100], [101], or is the water transport due solely to K^+^-channel function [102]? Regardless, this is a separate avenue of research and does not fulfill the scope of the present study. Taken together, caiman Müller glial cells appear to be unique among the vertebrates studied so far, as their hyperpolarized membrane potential and high K^+^ conductance, both essential for a wealth of glia-neuron interactions in the retina [3], [4], rely upon TASK 2P rather than Kir-type K^+^ channels.

### Coupling of Müller glial cells

Here we show that caiman Müller cells, similar to Müller cells of other species [9], [82], [103], [104], display somewhat limited coupling to their immediate cellular neighbors (Fig. 3C-F) and express the gap-junction protein, connexin 43 (Fig. 6). The Müller cell-Müller cell coupling may also contribute to the ‘passive’ I/V characteristic of the currents recorded in retinal wholemount preparations (Fig. 3B).

More strikingly, however, we observed a coupling between Müller cells and photoreceptor cells. This coupling appears to allow for a free bi-directional flux of small cations such as K^+^, as the electrophysiological properties recorded from enzymatically dissociated Müller cells were changed if the dissociation process failed to detach all photoreceptor cells from the Müller cell (Fig. 4). This coupling may be mediated by gap junctions formed by connexin 43 (Fig. 6). An expression of connexin 43 by Müller cells as well as neurons has been demonstrated before [105] but to the best of our knowledge, electrical coupling between Müller cells and photoreceptor cells is a novel finding. At present the functional role can only be speculated upon; however, it is tempting to suggest that spatial buffering of excess K^+^, one of the most fundamental functions of Müller cells [3] may involve direct K^+^ fluxes between Müller cells and photoreceptor cells in the caiman retina.

We also found evidence for an uni-directional coupling between cone photoreceptors and Müller cells, allowing for the transfer of the non-charged tracer, sulforhodamine-B, from cones to Müller cells but not vice versa (and not between adjacent cones) (Fig. 5). A uni-directional coupling has earlier been described between different types of glial cells [106], [107], [108], but not between glia and neurons in retina. Now it remains to be clarified how such uni-directional coupling is established. The observed heterologous cone-to-Müller cell coupling appears to be a unique novel finding, particularly as it does not involve homologous cone-to-cone coupling (Fig. 5A). This might be indicative of a hierarchy of signaling between neighboring cells, useful, for instance, for molecular filtering of large size molecules but not of small cations.

## Conclusion

This is the first functional study of retinal glial Müller cells from a representative of the crocodiles, the Spectacled caiman. The cells share many properties with Müller cells in other vertebrates but display several particular features, (1) a unique endowment of caiman Müller cells with K^+^ channels, substituting Kir-like K^+^ channels by TASK 2P channels; (2) electrical coupling of Müller glial cells with photoreceptors and (3) unidirectional tracer propagation from cones to Müller cells. It may be speculated that heterologous coupling between Müller cells and photoreceptor cells may allow for a specific cell-to-cell molecular signaling and modification of spatial buffering of K^+^ ions.

## Supporting Information

Figure S1 **Localization of the water channel AQP4 in the caiman retina.** (**A**) Immuno­staining for AQP4 (1∶200, Sigma-Aldrich A5971-primary antibody) followed by the secondary antibody carbocyanine Cy2-coupled donkey anti-rabbit IgG (green, 1∶200) revealed a strong signal in the inner limiting membrane (ILM). (**B**) Omitting the AQP4 antibody demonstrated autofluorescence in the outer retina. Cell nuclei were stained by Hoechst 33258 (blue). (**C**) Additional immuno-staining for AQP4 made in another caiman retina using a different AQP4 primary antibody (1∶200, Santa Cruz Biotechnology SC-20812) followed by the secondary antibody carbocyanine Cy3-coupled goat anti-rabbit IgG (red, 1∶200) revealed a strong labeling in the ILM, as well. GCL, ganglion cell layer; INL, inner nuclear layer; IPL, inner plexiform layer; ONL, outer nuclear layer; OPL, outer plexiform layer.
(TIF)

Figure S2 **Sulfonylurea-1 receptor (SUR1) subunit of KATP channels compared with reference markers in the caiman retinae.** (**A**) Nomarski image: no expression for SUR1 was found in Müller cells or in other caiman retinal cells. **B** and **C** show **no SUR1 staining** (bright contrast images) in different caiman retinae. (**D**) Other markers of Müller cells are shown to allow assessment of the cellular organization: lectin peanut agglutinin that is specific for cones (PNA, blue), glutamine synthetase in Müller cells (GS, red) and connexin-43 (Cx43, green) also specific for Müller cells. Scale bar  = 20 µm. ILM-inner limiting membrane, GCL-ganglion cell layer, IPL-inner plexiform layer, INL-inner nuclear layer, ONL-outer nuclear layer, IS-photoreceptor inner segments.
(TIF)
