# Supplementary figures and images for: Unidirectional Photoreceptor-to-Müller Glia Coupling and Unique K+ Channel Expression in Caiman Retina
Source: PLoS One. 2014 May 15;9(5):e97155. doi: 10.1371/journal.pone.0097155 (PMC4022631; doi:10.1371/journal.pone.0097155)

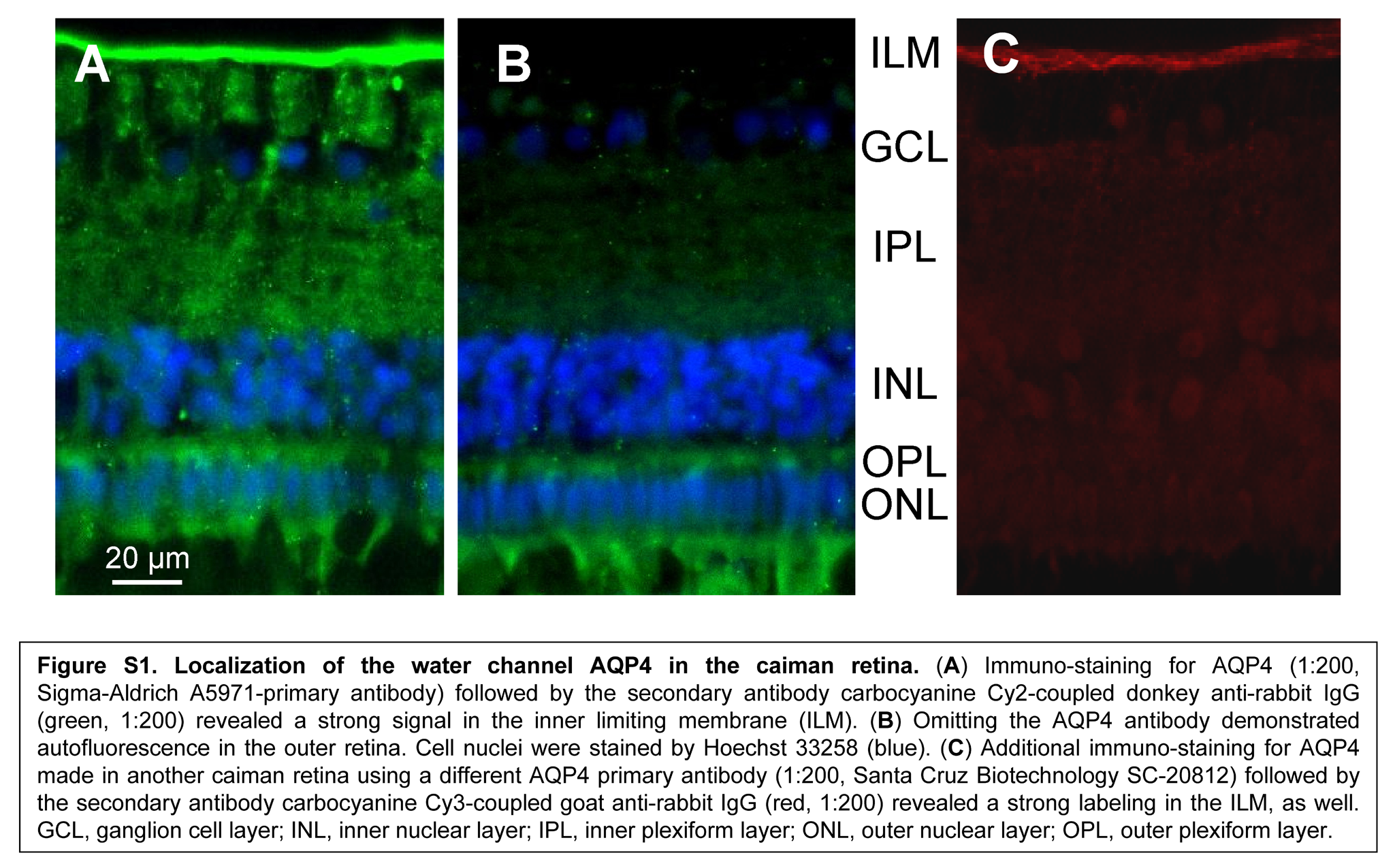

Supplement: Figure S1 — Localization of the water channel AQP4 in the caiman retina. (A) Immuno­staining for AQP4 (1∶200, Sigma-Aldrich A5971-primary antibody) followed by the secondary antibody carbocyanine Cy2-coupled donkey anti-rabbit IgG (green, 1∶200) revealed a strong signal in the inner limiting membrane (ILM). (B) Omitting the AQP4 antibody demonstrated autofluorescence in the outer retina. Cell nuclei were stained by Hoechst 33258 (blue). (C) Additional immuno-staining for AQP4 made in another caiman retina using a different AQP4 primary antibody (1∶200, Santa Cruz Biotechnology SC-20812) followed by the secondary antibody carbocyanine Cy3-coupled goat anti-rabbit IgG (red, 1∶200) revealed a strong labeling in the ILM, as well. GCL, ganglion cell layer; INL, inner nuclear layer; IPL, inner plexiform layer; ONL, outer nuclear layer; OPL, outer plexiform layer. (TIF) [file pone.0097155.s001.tif]

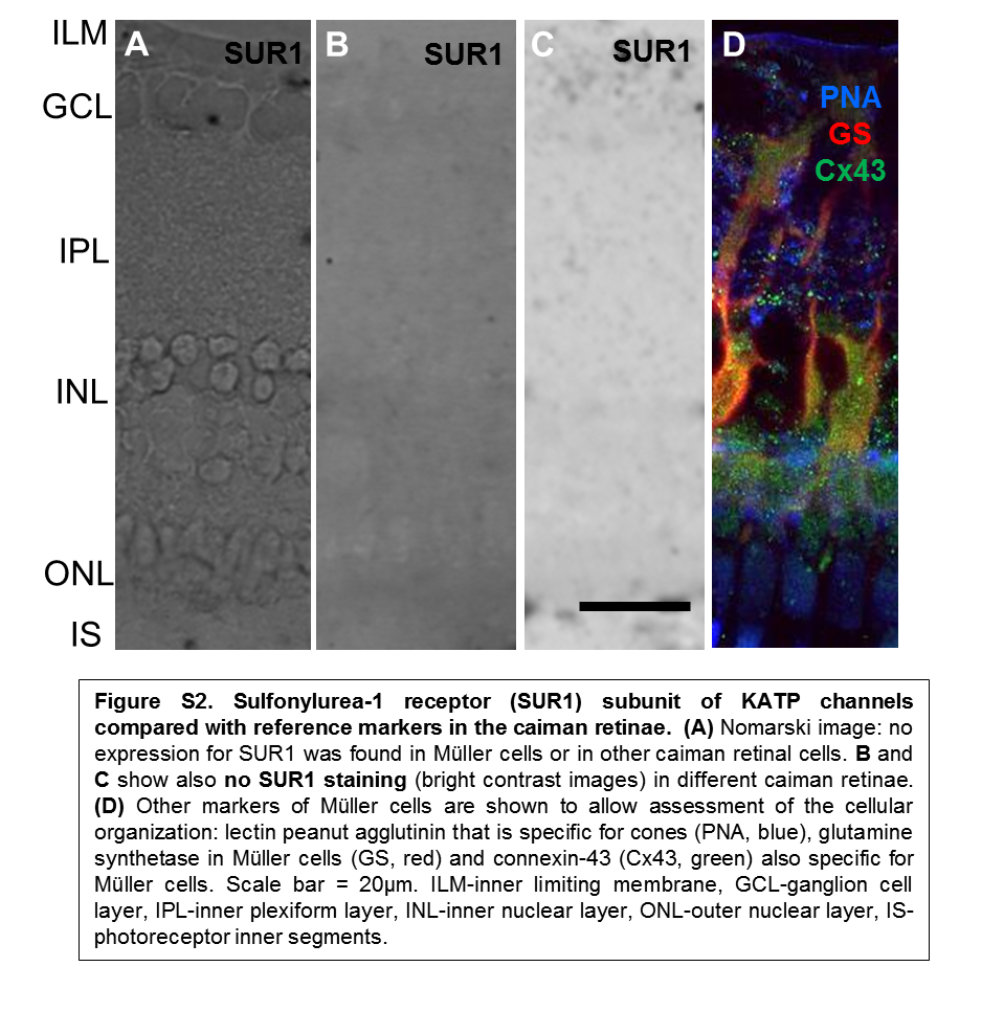

Supplement: Figure S2 — Sulfonylurea-1 receptor (SUR1) subunit of KATP channels compared with reference markers in the caiman retinae. (A) Nomarski image: no expression for SUR1 was found in Müller cells or in other caiman retinal cells. B and C show no SUR1 staining (bright contrast images) in different caiman retinae. (D) Other markers of Müller cells are shown to allow assessment of the cellular organization: lectin peanut agglutinin that is specific for cones (PNA, blue), glutamine synthetase in Müller cells (GS, red) and connexin-43 (Cx43, green) also specific for Müller cells. Scale bar = 20 µm. ILM-inner limiting membrane, GCL-ganglion cell layer, IPL-inner plexiform layer, INL-inner nuclear layer, ONL-outer nuclear layer, IS-photoreceptor inner segments. (TIF) [file pone.0097155.s002.tif]
